# Development of Single-Nucleotide Polymorphism (SNP)-Based Species-Specific Real-Time PCR Assays for Authenticating Five Highly Priced Tuna

**DOI:** 10.3390/foods13223692

**Published:** 2024-11-20

**Authors:** Meng Qu, Yanhua Jiang, Na Li, Yingying Guo, Wenjia Zhu, Na Li, Xinnan Zhao, Lin Yao, Lianzhu Wang

**Affiliations:** 1 Key Laboratory of Testing and Evaluation for Aquatic Product Safety and Quality, Ministry of Agriculture and Rural Affairs, Yellow Sea Fisheries Research Institute, Chinese Academy of Fishery Sciences, Qingdao 266071, China; qumeng@ysfri.ac.cn (M.Q.);; 2 Shandong Institute for Product Quality Inspection, Jinan 250102, China

**Keywords:** tuna, species identification, 2b-RAD, SNP, real-time PCR

## Abstract

Tuna are economically important as food resources in food markets. However, because tuna is often processed into steaks or fillets, the meat can be difficult to identify through morphological features. For effective fishery management and to protect the rights of consumers, it is necessary to develop a molecular method to accurately identify the species used in tuna products. Herein, we discovered five single-nucleotide polymorphism (SNP) sites via 2b-RAD sequencing and developed five SNP-based real-time polymerase chain reaction assays for the rapid identification of five highly priced tuna species. Three species-specific TaqMan systems were designed to identify albacore tuna (*Thunnus alalunga*), bigeye tuna (*T. obesus*), and southern bluefin tuna (*T. maccoyii*) and two cycling systems were designed to identify yellowfin tuna (*T. albacares*) and Atlantic bluefin tuna (*T. thynnus*). The systems showed good specificity and sensitivity (sensitivity of 0.0002 ng μL^−1^ for albacore tuna, bigeye tuna, and southern bluefin tuna and 0.002 ng μL^−1^ for yellowfin tuna and Atlantic bluefin tuna). Both systems were able to distinguish the target species from other species in a specific, sensitive, and accurate manner. Thus, these methods can be employed for the identification of species used in tuna products, protecting consumers and producers from economic fraud.

## 1. Introduction

The genus *Thunnus* includes some of the most economically important and thus most severely overexploited fish. The genus *Thunnus* belongs to the family Scombridae and comprises eight species, which are known as tuna [1,2]. Atlantic bluefin tuna (*Thunnus thynnus*), southern bluefin tuna (*T. maccoyii*), yellowfin tuna (*T. albacares*), bigeye tuna (*T. obesus*), and albacore tuna (*T. alalunga*) are generally of great economic value [3]. Species identification of tuna fish is important because different tuna species have very different prices, and it is typically based on morphological characteristics. However, identification of these species in their traded form is difficult due to them being traded in the form of chilled or frozen steaks or fillets. A recent survey of commercially available tuna products revealed some mislabeling, fraud, and substitution with cheaper fish [4,5]. For instance, one study [6] analyzed canned tuna and found that 24.4% of cans of yellowfin tuna were mislabeled. Another study detected tuna species substitution in 37% of cases at the point of sale and in 48% of cases at restaurants; the species substitution was mostly of bluefin tuna [7]. Seafood mislabeling poses health risks [8,9] and economic losses and impacts the normal business order of the market. Therefore, the identification of tuna species is becoming a topic of growing concern.

DNA-based methods for species identification tend to be reliable because of their robustness, high specificity, and sensitivity. Such methods can be used on almost all kinds of samples, including whole individual fish and processed fish products. Various polymerase chain reaction (PCR) assays for species identification of tuna have been reported previously, such as a multiplex PCR assay to identify the five common commercial tuna species (bigeye, skipjack, Atlantic bluefin, albacore, and yellowfin) [10]. Restriction fragment length polymorphisms (RFLPs) have also been used to differentiate between species of tuna [3,5,11]. However, these methods are technically complex and time-consuming, making them unsuitable for high-throughput examination.

More recently, real-time PCR has been used to successfully distinguish yellowfin tuna from skipjack tuna, Atlantic bonito, and bullet tuna [6]. However, the previous studies did not develop or describe any real-time PCR methods for distinguishing Atlantic bluefin tuna, southern bluefin tuna, yellowfin tuna, bigeye tuna, and albacore tuna, five high-value tuna species. Designing specific primers and probes of these tuna species is difficult due to the high homology and relatively high intraspecific variability between them [6,12]. Single-nucleotide polymorphisms (SNPs) are useful genetic markers for assigning species or populations and have been used in diverse ecological and biomedical studies [13] to study the phylogeny and evolution of closely related species [14,15,16]. Various methods have been employed to detect SNPs, including PCR-based, microarray, and DNA chip methods. Compared with other methods, PCR-based techniques are the most powerful to genotype candidate SNPs that can identify species rapidly at a low cost as they rely on standard reagents, equipment, and methods that are readily available in the laboratory [17,18]. Two-enzyme restriction-site-associated DNA sequencing (2b-RADseq) is a high-throughput genomic technique that involves the sequencing of the uniform fragments produced by type-IIB restriction endonucleases. 2b-RADseq produces high coverage of homologous SNP loci of fixed length [19], reduces genomic complexity [20], and is a useful method for genome-wide SNP discovery.

In this study, we aimed to identify sufficient novel SNPs in five high-value species tuna (Atlantic bluefin tuna, southern bluefin tuna, yellowfin tuna, bigeye tuna, and albacore tuna) via 2b-RADseq technology to develop a SNP-based real-time PCR assay that could specifically identify these species. The practical specificity and sensitivity of the five species assays were confirmed against collected samples. The method was employed on commercial food products to confirm applicability. The purpose of this study was to discriminate between tuna species via PCR and offer a rapid, accurate, and effective analysis method for the specific detection of the five high-value tuna species in commercially available tuna products, including raw tuna and sashimi, to make routine supervision feasible.

## 2. Materials and Methods

### 2.1. Samples

Atlantic bluefin tuna, southern bluefin tuna, yellowfin tuna, bigeye tuna, and albacore tuna were collected by our researchers or provided by Shandong Zhong Lu Oceanic Fisheries Co., Ltd. (Yantai, China). The fish were collected from different catching areas, including Atlantic, Indian, and Pacific Ocean tuna fisheries, and were morphologically identified according to the guidelines provided at Atuna.com (http://atuna.com/index.php/en/tuna-info/tuna-species-guide, accessed on 18 January 2017). Additionally, skipjack tuna (*Katsuwonus pelamis*) were collected for use as negative controls, and their identities were confirmed via a DNA barcoding method [21]. The information regarding the tuna samples is shown in Table 1.

A total of 70 commercially available prepackaged tuna products (raw and frozen) labeled as “Albacore tuna”, “Bigeye tuna”, “Yellowfin tuna”, “Bluefin tuna”, and “Tuna” were purchased from local supermarkets, seafood wholesale markets, sushi restaurants in Qingdao (Shandong, China), and e-commerce platforms. All samples were stored at −20 °C until their use in the experiments.

### 2.2. DNA Extraction

About 30 milligrams of fish muscle was cut and placed into a centrifuge tube. DNA was extracted from the samples according to the protocol described by Armani et al. [22]. The concentrations (in ng/mL) of DNA were assessed at 260 nm using a NanoPhotometer Pearl (Implen, München, Germany), and its integrity was evaluated by electrophoresis. The DNA extracts were stored at −20 °C until further analysis.

### 2.3. SNP Site Identification and Selection

The 2b-RAD libraries were prepared at Qingdao OE Biotech Co., Ltd. (Qingdao, China) as described by Wang et al. [23] with some modifications. In brief, 5 tuna fish were selected from each of the 11 currently hypothesized stocks from the Indian Ocean, the northern and southern parts of the Pacific Ocean, and the Atlantic Ocean, and a total of 103 fish were selected (Table 1). 2b-RAD genotyping was performed in the RAD typing program, and a high-quality reference was generated by in silico searching for 2b-RAD tags containing the restriction site of *BasXI* in the *T. orientalis* genome (https://www.ncbi.nlm.nih.gov/assembly/GCA_000418415.1/, accessed on 15 March 2017). The final library was sequenced on the Illumina PE sequencing platform. Enzyme reads were filtered using default parameters, and all SNP genotypes were obtained and mapped to reference sequences using SOAP [24]. The iML algorithm was adopted to exclude any repetitive sites from the genotyping [25]. Quality control was performed using the following criteria: minor allele frequency (MAF) > 0.05 and SNP call rate > 0.9 to exclude low-quality SNPs [26]. The following criteria were applied for SNP filtering for obtaining robust results: (1) polymorphic loci exhibiting more than two alleles were excluded [27]; (2) segregating markers that could be genotyped in more than 80% of the individuals were kept; (3) SNPs with a MAF of <0.01 were discarded; (4) only one biallelic SNP at each locus was retained [28]. The filtered SNPs were used for subsequent analyses.

### 2.4. PCR Amplification

#### 2.4.1. Design of PCR Primers and Probes

All sequences were based on the results of SNPs located in the chromosome. TaqMan probes were designed for ALB, BET, and SBT, and cycling probes were designed for YFT and BFT. The primers and probes were designed using Primer 5.0 and synthesized by Sangon Biotech Co., Ltd. (Shanghai, China). The sequences and sites of the primers and probes are shown in Table 2 and Table 3, respectively.

#### 2.4.2. TaqMan PCR Amplification

Real-time PCR was performed using the specific primers and probes designed for each species: ALB, BET, and SBT. The sequences of the primers and probes are detailed in Table 2. The real-time PCR was optimized and conducted in a total volume of 20.0 μL. Each reaction mixture contained 10 μL of 2×Premix Ex Taq (Probe qPCR) (Takara Bio Inc., Kusatsu, Japan), 0.4 μL (10 μmol μL^−1^) of primers and 0.2 μL (10 μmol μL^−1^) of probe specific for each tuna species, 3.0 μL of DNA extracts (20 ng~100 ng), and 6.0 μL of ddH_2_O. PCR was performed on a Roche LightCycler 480II System (Roche, Basel, Switzerland) under the following conditions: initial denaturation at 95 °C for 10 min; followed by 40 cycles of denaturation at 95 °C for 15 s and annealing at 60 °C for 1 min. In each assay, the experiments were conducted in triplicate. DNA extracted from skipjack tuna was used as the negative control, and sterile water was used as the blank control.

#### 2.4.3. Cycling Probe PCR Amplification

Real-time PCR was performed using the Cycleave PCR™ Starter Kit (Takara Bio Inc.) to identify YFT and BFT. The sequences of the primers and probes are shown in Table 3. The real-time PCR was optimized and conducted in a total volume of 25.0 μL. Each reaction mixture contained 12.5 μL of 2× Cycleave PCR Reaction Mixture, 0.5 μL (10 μmol μL^−1^) of primers and 0.5 μL (10 μmol μL^−1^) of probe specific for each tuna species, 2.0 μL of DNA extracts (20 ng~100 ng), and 9.0 μL of ddH_2_O. PCR was performed using a Roche LightCycler 480II System in three parallel experiments and the cycle conditions were as follows: initial denaturation at 95 °C for 30 s; followed by 45 cycles at 95 °C for 5 s for denaturation, annealing at 55 °C for 10 s, and a final extension step at 72 °C for 20 s. In each assay, DNA extracted from skipjack tuna was used as the negative control, and sterile water was used as the blank control.

#### 2.4.4. Sensitivity of the Real-Time PCR Assays

To estimate the sensitivities of each of the real-time PCR assays, DNA templates of ALB, BET, SBT, YFT, and BFT (20.0 ng μL^−1^) were diluted in a tenfold series (2.0, 0.2, 0.02, 0.002, 0.0002, and 0.00002 ng μL^−1^) in sterile ddH_2_O. The real-time PCR assays were performed as described in Section 2.4.2 and Section 2.4.3 with serial dilutions of the templates, and all tests were performed in triplicate. The amplification efficiency was calculated by the following equation: E = [10 ^(−1/slope)^ − 1] × 100 [29].

### 2.5. Commercial Tuna Product Test

The real-time PCR method was used to detect ALB, BET, YFT, BFT, and SBT in 70 commercial samples. The species of these tuna samples were confirmed using the DNA barcoding test developed by Ward et al. [21]. The primers for the DNA barcoding test are shown in Table 4.

## 3. Results and Discussion

### 3.1. SNP Markers of Five Tuna Species

This study reports the results of chromosome SNPs in BFT, SBT, YFT, BET, and ALB. Genotyping via the 2b-RAD method identified 68,464 SNPs from 103 samples. After quality control, a dataset of 3720 SNPs was yielded. By sequence comparison and filtering, 20 SNPs were selected manually from each tuna species, and 100 SNPs for five tuna species were discovered. The final selection in this study is shown in Table 5. SNPs are highly specific and sensitive to genetic variation, providing accurate discrimination and having widespread presence throughout the genome. SNPs have been used to identify genetic variation within and between populations or species and have become the preferred genetic markers for analyzing partially or fully sequenced genomes [30]. Previously, we have successfully established real-time PCR assays to distinguish Dissostichus [31], oilfish [32], and sablefish [33] based on variation sites in traditional genes concerning phylogenetic analysis, like Cyt*b*, COI, and *16SrRNA*, of the mitochondrial genome (mtDNA) successfully, but we failed to apply this technical route to tuna authentication. So far, most studies regarding the authentication of tuna have been based on the analysis of genetic markers obtained from the genes mentioned above in the mtDNA. However, SNPs that could completely discriminate between the five *Thunnus* species were difficult to determine in these traditional genes because of the high degree of homology among the DNA sequences of *Thunnus* species [34]. Another reason for this difficulty may be that these traditional genes are too short to find special sites and design primers/probes for species-specific PCR identification. Previous studies have developed real-time PCR assays for distinguishing only two or three species of tuna, likely for this underlying reason [34,35,36]. Therefore, we applied the 2b-RADseq technique to explore other SNPs for species-specific PCR identification of the whole genome of tuna.

### 3.2. Evaluation of the Primers and Probes for Real-Time PCR Assay

The fraudulent labeling of fish products is becoming increasingly prevalent because of the increasing demand for high-quality food. Real-time PCR demonstrates excellent efficiency, high sensitivity, and high specificity, while being without post-PCR steps that could decrease the risks of cross-contamination. Real-time PCR has therefore been widely used for the identification of species commonly mislabeled as tuna. Liu et al. [37] identified five tuna species (SBT, BET, YFT, ALB, and skipjack tuna) from processed and fresh tuna samples using a duplex quantitative real-time PCR (qPCR) assay based on the 16S rDNA gene; however, targets with Cq > 30 were not readily discriminated from nonspecific amplification. Therefore, a cycle cut-off point was set at 30, which is not the normal limit of detection in qPCR. Terio et al. [35] established real-time PCR assays to distinguish three tuna species commonly used for the production of canned tuna (YFT, BFT, and ALB) based on cytochrome *b* genes. Lopez and Pardo [36] developed real-time PCR assay based on mtDNA to identify two tuna species (ALB and YFT). However, mtDNA can relatively affect both the sensitivity and specificity of the reaction, as well as the quantitative measurement [38]. The methods described in the previous studies could not specifically and accurately detect or discriminate five highly priced tuna species, which are mostly traded as vacuum-packed slices or loin products. We developed five real-time PCR assays based on SNP markers located in the whole genome that were able to detect the five species of *Thunnus*. Concerning the number of species that could be identified, the methods developed in this study are more in line with the practical needs of high-value tuna and its raw product authentication.

#### 3.2.1. TaqMan Real-Time PCR Assay for ALB, BET, and SBT

**Figure 1:**
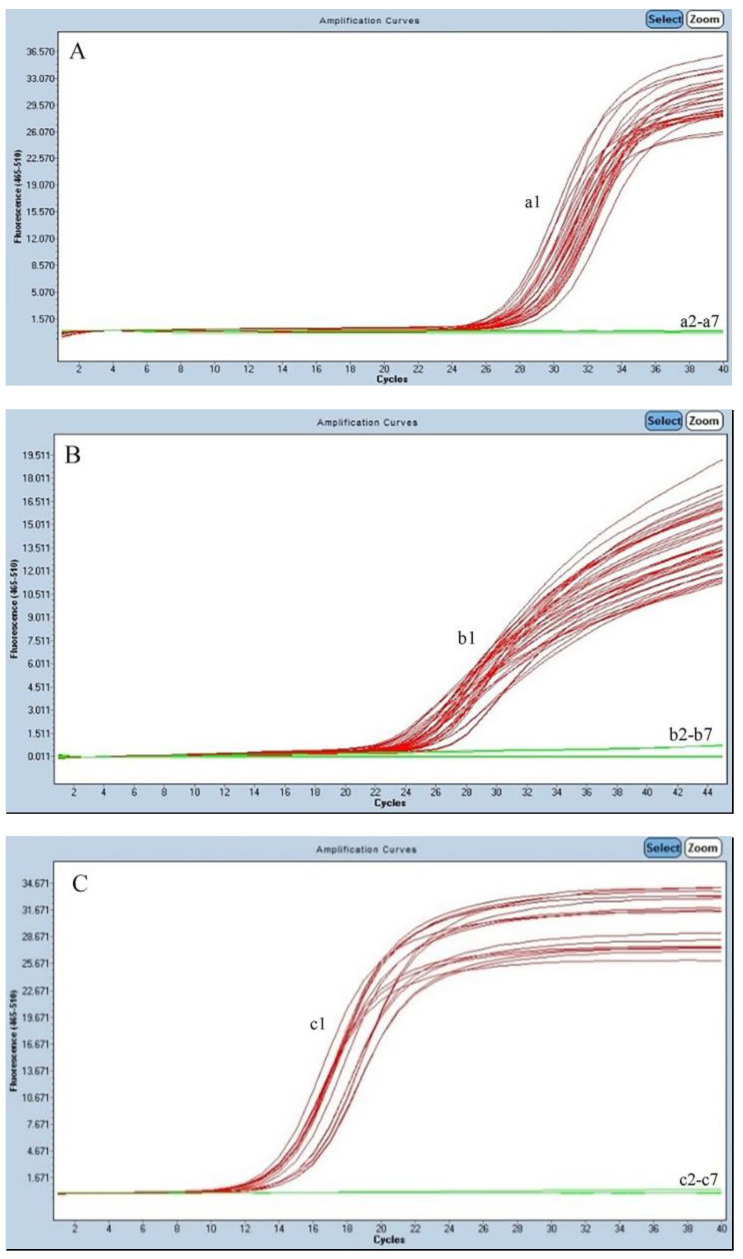

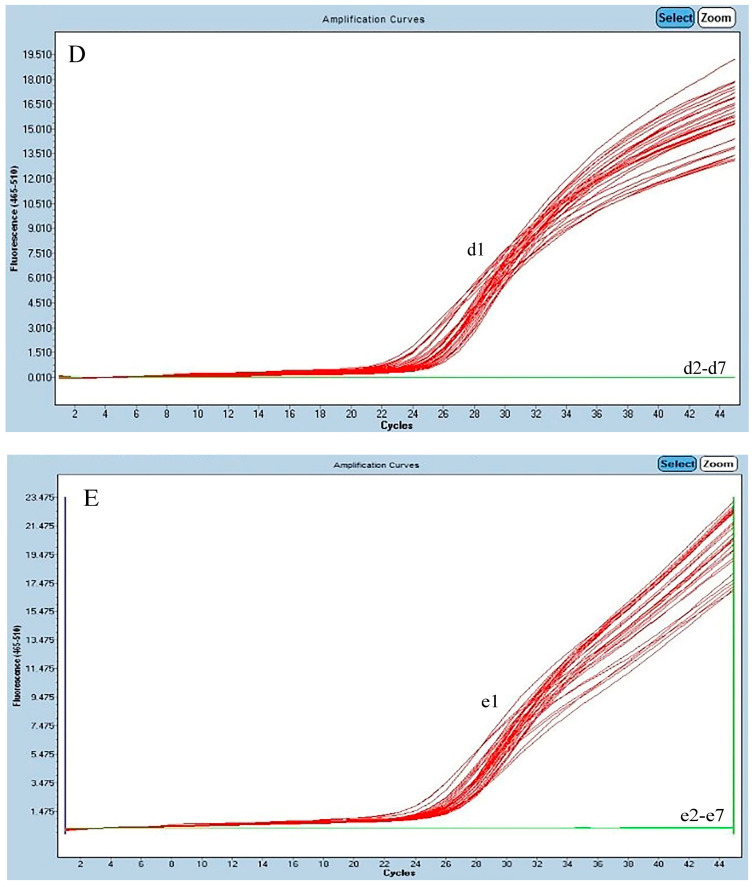
Specific analysis for the real-time PCR method to detect SBT (**A**), BET (**B**), ALB (**C**), BFT (**D**), and YFT (**E**). (**A**). Primers and probes SBT 07 for SBT. (a1) SBT; (a2–a5) ALB, BET, YFT, BFT; (a6) NC; (a7) Blank control. (**B**). Primers and probes BET 81 for BET. (b1) BET; (b2–b5) ALB, SBT, YFT, BFT; (b6) NC; (b7) Blank control. (**C**). Primers and probes ALB 66 for ALB. (c1) ALB; (c2–c5) BET, SBT, YFT, BFT; (c6) NC; (c7) Blank control. (**D**). Primers and probes BFT 24 for YFT. (d1) YFT; (d2–d5) ALB, BET, BFT, SBT; (d6) NC; (d7) Blank control. (**E**). Primers and probes YFT 43 for BFT. (e1) BFT; (e2–e5) BET, SBT, ALB, YFT; (e6) NC; (e7) Blank control.

The specificity of the primers was confirmed by real-time PCR using a total of five tuna species (103 samples, described in Section 2.1). The TaqMan probe assays are highly specific since no cross-reactions were observed among the five tuna species in the three TaqMan systems (Figure 1). The TaqMan probe assays specifically amplified the DNA of ALB, BET, and SBT in the samples successfully detected. “S-shaped” positive amplification curves were observed in the target DNA templates, showing an average quantification cycle (Cq) value (±SD) of 25.37 ± 0.36 for SBT, 23.68 ± 0.25 for BET, and 14.39 ± 0.45 for ALB. DNA templates from the other tuna samples or the blank control did not amplify after 40 cycles. In previous studies, the amplification curves from real-time PCR showed cross-reactions among the ALB, BET, YFT, BFT, and SBT. Therefore, the ΔCts and fluorescence endpoint values of the experimental system had to be used as criteria for identification [34,38], making the assay complicated. The probe of the TaqMan real-time PCR assay avoids nonspecific amplification, hybridizes specifically only with the target sequence to further improve the accuracy of the results and amplify small products, and is precise and sensitive to low template DNA concentration [39,40].

#### 3.2.2. Identification of YFT and BFT by Cycling Systems

In this study, TaqMan primer probes targeting YFT and BFT were developed; however, unexpected cross-reactions were observed among SBT, BET, and ALB. The Cycleave PCR method can be applied to identify SNPs and strictly distinguish a single-base mutation [41]. Therefore, we designed two cycling probes based on the SNPs for real-time PCR detection that could identify YFT and BFT. The Cycleave PCR method established specifically amplified the DNA of YFT and BFT (Figure 1), showing average Cq values (±SD) of 23.37 ± 0.34 for YFT and 23.42 ± 0.32 for BFT. The other fish samples and the blank control did not amplify after 40 cycles. This method showed high specificity and sensitivity in detecting SNPs which were comparable to those of the TaqMan method. In the analysis of single-nucleotide variation, the *Tm* difference between the sequences is low [42], which could be the reason for the cross-reactions between SBT, BET, and ALB with the TaqMan method, even when the annealing temperature was optimized. The Cycleave PCR method amplifies the template using a pair of primers and a chimeric DNA–RNA–DNA probe. PCR-amplified DNA generates a complete hybrid with the RNA portion of the mutant probe when mutant molecules are present [43] and showed extreme specificity and enhanced accuracy. As a result of this modification, the oligonucleotide was able to bind to its target with excellent specificity and accuracy [44].

### 3.3. Sensitivity Evaluation

**Figure 2:**
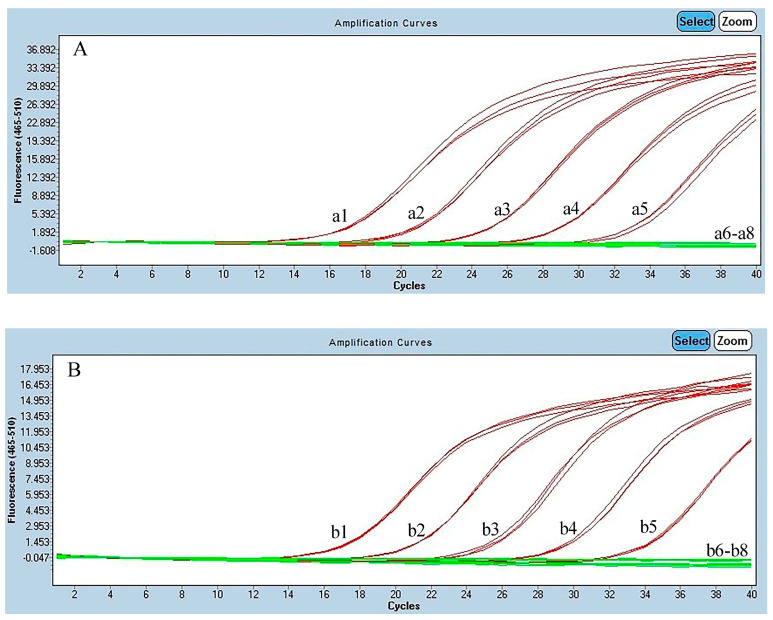

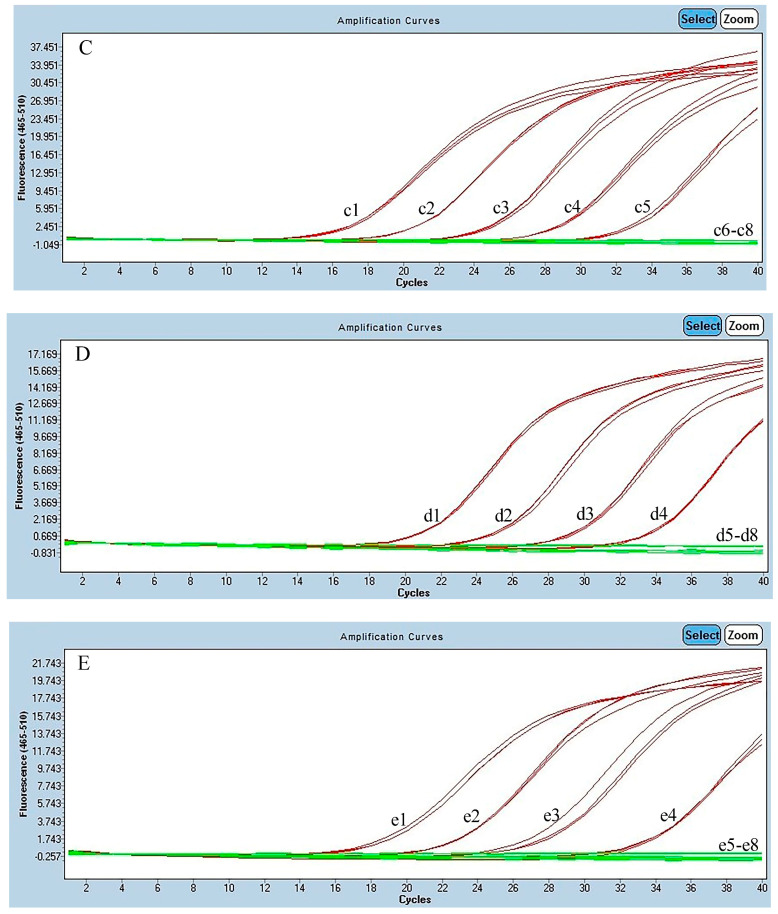
The sensitivity of real-time polymerase chain reaction (PCR) for BFT, SBT, YFT, BET, and ALB detection. (**A**) SBT real-time PCR. (a1–a6) The concentrations of the DNA templates for SBT detection were 2, 0.2, 0.02, 0.002, 0.0002, and 0.00002 ng μL^−1^, respectively; (a7) NC; (a8) Blank control. (**B**) BET real-time PCR. (b1–b6) The concentration of the DNA templates for BET detection were 2, 0.2, 0.02, 0.002, 0.0002, and 0.00002 ng μL^−1^, respectively; (b7) NC; (b8) Blank control. (**C**) ALB real-time PCR. (c1–c6) The concentrations of the DNA templates for ALB detection were 2, 0.2, 0.02, 0.002, 0.0002, and 0.00002 ng μL^−1^, respectively; (c7) NC; (c8) Blank control. (**D**) YFT real-time PCR. (d1–d6) The concentrations of the DNA templates for YFT detection were 2, 0.2, 0.02, 0.002, 0.0002, and 0.00002 ng μL^−1^, respectively; (d7) NC; (d8) Blank control. (**E**) BFT real-time PCR. (e1–e6) The concentrations of the DNA template for BFT detection were 2, 0.2, 0.02, 0.002, 0.0002, and 0.00002 ng μL^−1^, respectively; (e7) NC; (e8) Blank control.

**Figure 3:**
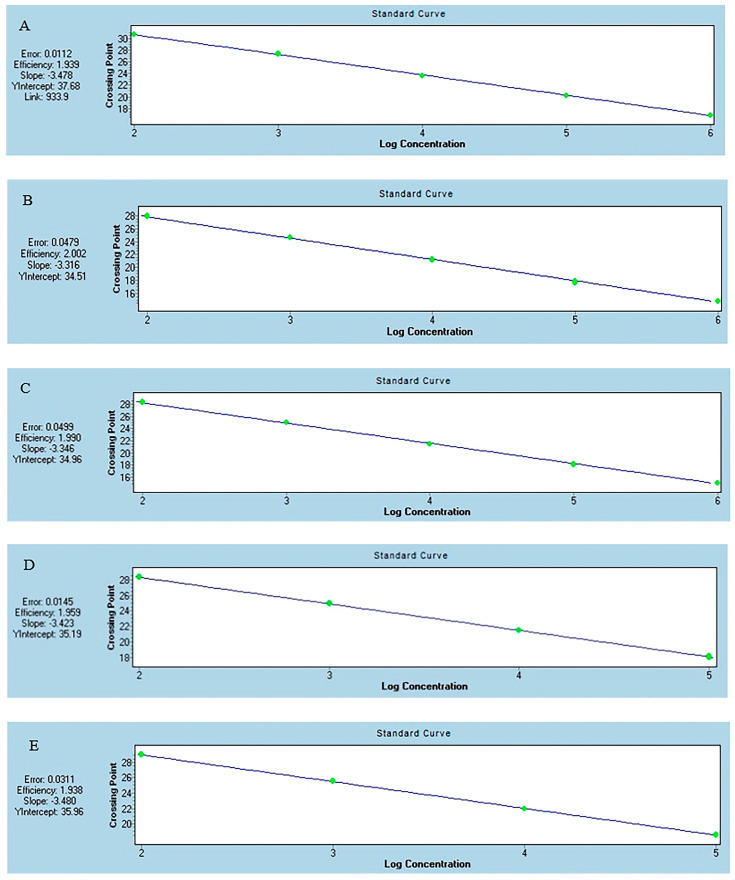
The amplification efficiency of the real-time polymerase chain reaction for SBT (**A**), BET (**B**), ALB (**C**), YFT (**D**), and BFT (**E**).

The real-time PCR detection of the DNA of BFT, SBT, YFT, BET, and ALB at various concentrations was evaluated. The LODs of DNA by these methods were 0.0002 ng μL^−1^ for ALB, BET, and SBT and 0.002 ng μL^−1^ for YFT and BFT (Figure 2). The sensitivity of the method for the cycling probe was lower than that for the TaqMan probe; however, all of them were able meet the needs of actual detection. The detection limits and amplification efficiencies of these methods differed slightly, which may have been caused by sporadic nucleotide mismatches in the primers and/or probes [45]. A higher *Tm* can stabilize the probe–template hybrid, whereas a lower *Tm* can cause low binding of the probe during the annealing phase which may be the reason for the low LOD [29]. The R^2^ values of these methods were >0.99, suggesting a good correlation between the quantification cycle values and the DNA concentration, indicating that this method has good anti-interference ability. The amplification efficiency was 93.91% for the SBT-specific method, 100.25% for the BET method, 99.05% for the ALB method, 95.91% for the YFT method, and 93.83% for the BFT method, all of which were within the optimal range of 90% to 110% [29], suggesting the high performance of the assay (Figure 3).

### 3.4. Commercial Fish Product Identification

Considering local consumption habits and high prices in the market, tuna, especially bluefin tuna, YFT, and BET, are rarely processed into grilled or canned products in China. Therefore, the applicability of the methods developed in this study was examined using raw steak or fillet samples, which had potentially been substituted or mislabeled. The real-time PCR methods were employed to identify the species of 70 product samples and we assessed the feasibility and accuracy of the application of this method to commercial prepackaged products which were further confirmed via DNA barcoding (Table S1). The results showed that among the 25 samples labeled as “Tuna”, 10 were identified as ALB, 6 were identified as YFT, and 9 produced negative results via real-time PCR but were identified as skipjack tuna using the DNA barcoding method. Although skipjack tuna belongs to the genus *Katsuwonus*, not *Thunnus*, this fish is considered as tuna by some countries and international organizations. However, in the Chinese domestic market, most traditional consumers or traders do not consider *Katsuwonus* as tuna; therefore, the price of skipjack tuna is much lower than that of *Thunnus* tuna. It seems that these nine skipjack tuna samples have the possibility of being economically motivated adulteration (EMA) for local consumers. However, in China, there are no laws or regulations that clearly declare that skipjack tuna is not real tuna. This confusing situation could be resolved by a mandatory requirement that the fish species information must be clearly labeled in future. Among the ten samples labeled as BET, the real-time PCR results identified nine samples as BET and the other sample as ALB. The latter sample may have been the result of intentional mislabeling as BET has a higher value than ALB in China. Out of the five samples labeled as ALB, the real-time PCR results identified all as ALB. Among the 14 samples labeled as YFT, the real-time PCR results identified 13 samples as YFT and the other sample as BET. This situation may have been caused by unintentional mislabeling as BET carries a higher price than that of YFT. Among the 16 samples labeled as bluefin tuna, the real-time PCR results identified 8 samples as BFT, 6 samples as SBT, and 2 samples as YFT. This may have been due to intentional mislabeling as the price of bluefin tuna is much higher than that of YFT.

Collectively, the results showed that the species identified by real-time PCR were consistent with the sequencing results, demonstrating the accuracy of the methods developed in this study. Seafood fraud is a kind of EMA, defined as “seafood sold under a name other than its true name”, and includes mislabeling (i.e., incorrect labeling of a product with another name) and substitution (i.e., replacing a product with a different product without informing) [17]. The occurrence of high-value bluefin tuna being replaced with lower-value tuna has been reported in previous studies [46], and a similar situation was also found in our data. The current situation of tuna EMA is better than it was previously due to the long-term efforts of Chinese market supervision. Most of the mislabeled samples detected in this study were collected from e-commerce platforms, where the species information is often ambiguous or false, especially for products whose prices deviate significantly from the normal price. This situation requires stricter regulation and supervision by authorities in the future.

## 4. Conclusions

Our data suggest that tuna species can be identified via genome-wide SNPs using 2b-RAD sequencing. Five SNP loci were identified in this study and specific primers and probes were designed based on the five SNP loci of ALB, SBT, YFT, BET, and ALB. The developed methods showed excellent quality in terms of sensitivity, specificity, and application to commercial products. Therefore, these methods provide the technology needed to support the rapid identification of tuna species for quality inspections and scientific research. Moreover, the developed approach may prove particularly beneficial for rapid import and export inspection and onboard species identification to protect producers and consumers from economic fraud and could help protect tuna from overexploitation.

## Figures and Tables

**Table 1:** Sample information.

Latin Name	English Name	Abbreviation in This Study	Sampling Location	Sample Quantity
*T. alalunga*	Albacore tuna	ALB	The South Pacific Ocean	9
			The North Pacific Ocean	10
*T. obesus*	Bigeye tuna	BET	The Atlantic Ocean	10
			The Pacific Ocean	10
			The Indian Ocean	4
*T. albacares*	Yellowfin tuna	YFT	The Atlantic Ocean	10
			The Pacific Ocean	10
			The Indian Ocean	10
*T. thynnus*	Atlantic bluefin tuna	BFT	The Atlantic Ocean	10
*T. maccoyii*	Southern bluefin tuna	SBT	The Pacific Ocean	10
			The Indian Ocean	10

**Table 2:** Primers and TaqMan probes used in the real-time PCR system.

Name	Sequence (5′–3′)	Product Length	Purpose
SBT 07F	CCACAACCTCTGAGTCTGAACCT	114 bp	SBT identification
SBT 07R	GCAAAGGCTGATAGTAAACAACAAAT		
SBT 07P	FAM-TTTCATTCTGCCACTGTG-MGB		
ALB 66F	TCTCCATATTCATACTCCCATTGTCT	80 bp	ALB identification
ALB 66R	CTCTGCACATCCCTATTACCTACACA		
ALB 66P	FAM-AAACCATTCCTCCTTTGA-MGB		
BET 81F	GAGGGCAAAAAAAAGCCATTG	144 bp	BET identification
BET 81R	AGGTACCTGAGAGAGTAGCACATGTAGTA		
BET 81P	FAM-CCTGTCTCAATTAC-MGB		

**Table 3:** Primers and cycling probes used in the real-time PCR system.

Name	Sequence (5′–3′)	Product Length	Purpose
BFT 24F	GGAGGCACATACACTCATGAAACA	158 bp	BFT identification
BFT 24R	CTCAGTATCATCCCATGATGAACAA		
BFT 24P	FAM-CCTGGA(***A***)ACA-Eclipse		
YFT 43F	GAGTTGTGATGCTTACATT	109 bp	YFT identification
YFT 43R	TATCAGTGGTACAAGAGC		
YFT 43P	FAM-CACACATA(***G***)TA-BHQ1		

The italic boldface letters in the sequences of the probes BFT 24P and YFT 43P indicate a nucleotide that is replaced by RNA.

**Table 4:** Sequence of the primers for DNA barcoding.

Name	Sequence (5′–3′)	Product Length	Purpose
F1	TCAACCAACCACAAAGACATTGGCAC	∼655 bp	DNA barcoding test [21]
F2	TCGACTAATCATAAAGATATCGGCAC		
R1	TAGACTTCTGGGTGGCCAAAGAATCA		
R2	ACTTCAGGGTGACCGAAGAATCAGAA		

**Table 5:** SNP loci selected from 5 tuna species.

Tuna	Ref ID	Sequence	SNP Site Base
SBT	ref-345123	CATTCTGTCACTGTGTCTCCAAAAGGG	T
BFT	ref-152929	GCCTCATCTACATCCTCTCCAGTTAGT	T
YFT	ref-554392	ACACATAATACAACTCCTCCTTGAAAT	A
ALB	ref-159729	ATTTCAAAAACCATTCCTCCTCTGATT	C
BET	ref-170863	CAGTTACCTACATATACTCCTACTGTA	G

The boxed letters in the sequences indicate SNP location.

## Data Availability

The original contributions presented in the study are included in the article and Supplementary Material, further inquiries can be directed to the corresponding author.

## Supplementary Materials

The following supporting information can be downloaded at: https://www.mdpi.com/article/10.3390/foods13223692/s1, Table S1: Results of identification by real-time PCR and sequencing analysis for 70 commercial samples.

## References

[1] Díaz-Arce N., Arrizabalaga H., Murua H., Irigoien X., Rodríguez-Ezpeleta N. (2016). RAD-seq derived genome-wide nuclear markers resolve the phylogeny of tunas. Mol. Phylogenet. Evol..

[2] Vinas J., Tudela S. (2009). A validated methodology for genetic identification of tuna species (genus *Thunnus*). PLoS ONE.

[3] Yao L., Lu J., Qu M., Jiang Y., Li F., Guo Y., Wang L., Zhai Y. (2020). Methodology and application of PCR-RFLP for species identification in tuna sashimi. Food Sci. Nutr..

[4] Chen S., Zhang Y., Li H., Wang J., Chen W., Zhou Y., Zhou S. (2014). Differentiation of fish species in Taiwan Strait by PCR-RFLP and lab-on-a-chip system. Food Control.

[5] Mata W., Chanmalee T., Punyasuk N., Thitamadee S. (2020). Simple PCR-RFLP detection method for genus- and species-authentication of four types of tuna used in canned tuna industry. Food Control.

[6] Servusova E., Piskata Z. (2021). Identification of Selected Tuna Species in Commercial Products. Molecules.

[7] Gordoa A., Carreras G., Sanz N., Viñas J. (2017). Tuna Species Substitution in the Spanish Commercial Chain: A Knock-On Effect. PLoS ONE.

[8] Williams M., Hernandez-Jover M., Shamsi S. (2020). Fish substitutions which may increase human health risks from zoonotic seafood borne parasites: A review. Food Control.

[9] Roungchun J.B., Tabb A.M., Hellberg R.S. (2022). Identification of tuna species in raw and processed products using DNA mini-barcoding of the mitochondrial control region. Food Control.

[10] Lee G., Suh S., Lee Y., Kim H. (2022). Multiplex PCR Assay for Simultaneous Identification of Five Types of Tuna (Katsuwonus pelamis; Thunnus alalonga; T. albacares; T. obesus and T. thynnus). Foods.

[11] Abdullah A., Rehbein H. (2016). The differentiation of tuna (family: *Scombridae*) products through thePCR-based analysis of the cytochrome *b* gene and parvalbumin introns. J. Sci. Food Agric..

[12] Ceruso M., Mascolo C., Luca P.D., Venuti I., Biffali E., Ambrosio R.L., Smaldone G., Sordino P., Pepe T. (2021). Dentex dentex Frauds: Establishment of a New DNA Barcoding Marker. Foods.

[13] Kratochwil C.F., Kautt A.F., Rometsch S.J., Meyer A. (2022). Benefits and limitations of a new genome-based PCR-RFLP genotyping assay (GB-RFLP): A SNP-based detection method for identification of species in extremely young adaptive radiations. Ecol. Evol..

[14] Razkin O., Sonet G., Breugelmans K., Madeira M.J., Gómez-Moliner B.J., Backeljau T. (2016). Species limits; interspecific hybridization and phylogeny in the cryptic land snail complex Pyramidula: The power of RADseq data. Mol. Phylogenet. Evol..

[15] Leache A.D., Banbury B.L., Felsenstein J., de Oca A.N., Stamatakis A. (2015). Short Tree; Long Tree; Right Tree; Wrong Tree: New Acquisition Bias Corrections for Inferring SNP Phylogenies. Syst. Biol..

[16] Rubin B.E.R., Ree R.H., Moreau C.S., Kolokotronis S. (2012). Inferring phylogenies from RAD sequence data. PLoS ONE.

[17] Jin S.B., Kim H.B., Park S., Kim M.J., Choi C.W., Yun S. (2020). Identification of the ‘Haryejosaeng’ mandarin cultivar by multiplex PCR-based SNP genotyping. Mol. Biol. Rep..

[18] Niciura S.C.M., Cruvinel G.G., Moraes C.V., Bressani F.A., Malagó Junior W., Benavides M.V., de Chagas A.C. (2018). PCR-based genotyping of SNP markers in sheep. Mol. Biol. Rep..

[19] Luo X., Shi X., Yuan C., Ai M., Ge C., Hu M., Feng Y. (2017). Genome-wide SNP analysis using 2b-RAD sequencing identifies the candidate genes putatively associated with resistance to ivermectin in *Haemonchus contortus*. Parasites Vectors.

[20] Cui Z., Zhang J., Sun Z., Liu B., Zhao C., Chang Y. (2021). Identification of Sex-Specific Markers Through 2b-RAD Sequencing in the Sea Urchin (*Mesocentrotus nudus*). Front. Genet..

[21] Ward R.D., Holmes B.H., White W.T., Last P.R. (2008). DNA barcoding Australasian chondrichthyans: Results and potential uses in conservation. Mar. Freshw. Res..

[22] Armani A., Castigliego L., Tinacci L., Gianfaldoni D., Guidi A. (2011). Molecular characterization of icefish; (*Salangidae* family); using direct sequencing of mitochondrial cytochrome *b* gene. Food Control.

[23] Wang S., Liu P., Lv J., Li Y., Cheng T., Zhang L., Xia Y., Sun H., Hu X., Bao Z. (2016). Serial sequencing of isolength RAD tags for cost-efficient genome-wide profiling of genetic and epigenetic variations. Nat. Protoc..

[24] Li R., Li Y., Kristiansen K., Wang J. (2008). SOAP: Short oligonucleotide alignment program. Bioinformatics.

[25] Fu X., Dou J., Mao J., Su H., Jiao W., Zhang L., Hu X., Huang X., Wang S., Bao Z. (2013). RAD typing: An integrated package for accurate de novo codominant and dominant RAD genotyping in mapping populations. PLoS ONE.

[26] Dai P., Luan S., Lu X., Luo K., Cao B., Meng X., Kong J. (2017). Genetic evaluation of feed efficiency in the breeding population of *Fenneropenaeus chinensis* “Huanghai No. 2” using phenotypic; pedigree and genomic information. Aquac. Int..

[27] Xu T., Sun J., Lv J., Kayama Watanabe H., Li T., Zou W., Greg W.R., Wang S., Qian P., Bao Z. (2017). Genome-wide discovery of single nucleotide polymorphisms (SNPs) and single nucleotide variants (SNVs) in deep-sea mussels: Potential use in population genomics and cross-species application. Deep Sea Res. Part II Top. Stud. Oceanogr..

[28] Mu X., Sun M., Yang P., Lin Q. (2017). Unveiling the Identity of Wenwan Walnuts and Phylogenetic Relationships of Asian Juglans Species Using Restriction Site-Associated DNA-Sequencing. Front. Plant Sci..

[29] Raymaekers M., Smets R., Maes B., Cartuyvels R. (2009). Checklist for optimization and validation of real-time PCR assays. J. Clin. Lab. Anal..

[30] Yoo E., Haile M., Ko H., Choi Y., Cho G., Woo H., Wang X., Sung P., Lee J., Lee J. (2023). Development of SNP markers for Cucurbita species discrimination. Sci. Hortic..

[31] Yao L., Qu M., Jiang Y., Guo Y., Li N., Li F., Tan Z., Wang L. (2022). The development of genus-specific and species-specific real-time PCR assays for the authentication of Patagonian toothfish and Antarctic toothfish in commercial seafood products. J. Sci. Food Agric..

[32] Yao L., Xin H., Qu M., Jiang Y., Guo Y., Li F., Li N., Tan Z., Wang L. (2021). Development of duplex real-time polymerase chain reaction for simultaneous detection of oilfish- and escolar-derived components. J. Sci. Food Agric..

[33] Qu M., Jiang Y., Guo Y., Zhu W., Liu S., Li N., Li F., Tan Z., Wang L. (2023). Development and Evaluation of Real-Time Polymerase Chain Reaction (PCR) to Authenticate Sablefish (*Anoplopoma fimbria*) in Commercial Seafood Products. J. Aquat. Food Prod. Technol..

[34] Chuang P., Chen M., Shiao J. (2012). Identification of tuna species by a real-time polymerase chain reaction technique. Food Chem..

[35] Terio V., Di Pinto P., Decaro N., Parisi A., Desario C., Martella V., Buonavoglia C., Tantillo M. (2010). Identification of tuna species in commercial cans by minor groove binder probe real-time polymerase chain reaction analysis of mitochondrial DNA sequences. Mol. Cell. Probes.

[36] Lopez I., Pardo M.A. (2005). Application of Relative Quantification TaqMan Real-Time Polymerase Chain Reaction Technology for the Identification and Quantification of *Thunnus alalunga* and *Thunnus albacares*. J. Agric. Food Chem..

[37] Liu S., Xu K., Wu Z., Xie X., Feng J. (2016). Identification of five highly priced tuna species by quantitative real-time polymerase chain reaction. Mitochondrial DNA Part A.

[38] Mohamad N.A., El Sheikha A.F., Mustafa S., Mokhtar N.F.K. (2013). Comparison of gene nature used in real-time PCR for porcine identification and quantification: A review. Food Res. Int..

[39] Chen W., Fu Y., Zeng Z., Guo S., Yan Y., Tu Y., Tu Y., Gou T., Zhang Q. (2022). Establishment and application of a TaqMan probe–based qPCR for the detection of *Enterocytozoon hepatopenaei* in shrimp *Litopenaeus vannamei*. Parasitol. Res..

[40] O’Neill D., Turner P.D., O’Meara D.B., Chadwick E.A., Coffey L., O’Reilly C. (2013). Development of novel real-time TaqMan^®^PCR assays for the species and sex identification of otter (*Lutra lutra*) and their application to noninvasive genetic monitoring. Mol. Ecol. Resour..

[41] Wen J., Gou H., Liu J., Zhou H., Lin Q., Qu X., Chen K., Wang S., Shen H., Liao M. (2021). A one-step closed-tube enzyme-activated blocked probe assay based on SNP for rapid detection of *Salmonella pullorum*. Poult. Sci..

[42] Ishige T., Itoga S., Matsushita K. (2018). Locked Nucleic Acid Technology for Highly Sensitive Detection of Somatic Mutations in Cancer. Adv. Clin. Chem..

[43] Hou Y., Luo Q., Chen C., Zhou M. (2011). Application of cycleave PCR to the detection of a point mutation (F167Y) in the β 2 -tubulin gene of *Fusarium graminearum*. Pest Manag. Sci..

[44] Nan W., Zhang Y., Tan P., Xu Z., Chen Y., Mao K., Chen Y. (2016). A rapid cycleave PCR method for distinguishing the vaccine strainBrucella abortus A19 in China. J. Vet. Diagn. Investig..

[45] Suzuki Y., Saito R., Zaraket H., Dapat C., Caperig-Dapat I., Suzuki H. (2010). Rapid and specific detection of amantadine-resistant influenza A viruses with a Ser31Asn mutation by the cycling probe method. J. Clin. Microbiol..

[46] Pardo M.Á., Jiménez E., Viðarsson J.R., Ólafsson K., Ólafsdóttir G., Daníelsdóttir A.K., Begoña P.V. (2018). DNA barcoding revealing mislabeling of seafood in European mass caterings. Food Control.

