# Supplementary material for: Development of Single-Nucleotide Polymorphism (SNP)-Based Species-Specific Real-Time PCR Assays for Authenticating Five Highly Priced Tuna
Source: Foods. 2024 Nov 20;13(22):3692. doi: 10.3390/foods13223692 (PMC11593726; doi:10.3390/foods13223692)
Supplement: Supplementary file 1 [file foods-13-03692-s001.zip › foods-3275763-supplementary.pdf]

Table S1. Results of identification by real-time PCR and sequencing analysis for 70 commercial samples.

Sample Number	Sample Nominal	Sequence Analysis	Real-time PCR
sample-1*	Tuna	*K. pelamis*	-
sample-2*	Tuna	*K. pelamis*	-
sample-3	Tuna	*T. alalunga*	*T. alalunga*
sample-4	Tuna	*T. alalunga*	*T. alalunga*
sample-5	Tuna	*T. albacares*	*T. albacares*
sample-6*	Tuna	*K. pelamis*	-
sample-7	Tuna	*T. alalunga*	*T. alalunga*
sample-8	Tuna	*T. alalunga*	*T. alalunga*
sample-9	Tuna	*T. alalunga*	*T. alalunga*
sample-10	Tuna	*T. alalunga*	*T. alalunga*
sample-11*	Tuna	*K. pelamis*	-
sample-12	Tuna	*T. albacares*	*T. albacares*
sample-13	Tuna	*T. albacares*	*T. albacares*
sample-14	Tuna	*T. alalunga*	*T. alalunga*
sample-15	Tuna	*T. alalunga*	*T. alalunga*
sample-16*	Tuna	*K. pelamis*	-
sample-17*	Tuna	*K. pelamis*	-
sample-18	Tuna	*T. alalunga*	*T. alalunga*
sample-19	Tuna	*T. alalunga*	*T. alalunga*
sample-20*	Tuna	*K. pelamis*	-
sample-21	Tuna	*T. albacares*	*T. albacares*
sample-22	Tuna	*T. albacares*	*T. albacares*
sample-23*	Tuna	*K. pelamis*	-
sample-24*	Tuna	*K. pelamis*	-
sample-25	Tuna	*T. albacares*	*T. albacares*
BET-1	Bigeye tuna	*T. obesus*	*T. obesus*
BET-2	Bigeye tuna	*T. obesus*	*T. obesus*
BET-3	Bigeye tuna	*T. obesus*	*T. obesus*
BET-4	Bigeye tuna	*T. obesus*	*T. obesus*
BET-5	Bigeye tuna	*T. obesus*	*T. obesus*
BET-6*	Bigeye tuna	*T. alalunga*	*T. alalunga*
BET-7	Bigeye tuna	*T. obesus*	*T. obesus*
BET-8	Bigeye tuna	*T. obesus*	*T. obesus*
BET-9	Bigeye tuna	*T. obesus*	*T. obesus*
BET-10	Bigeye tuna	*T. obesus*	*T. obesus*
ALB-1	Albacore tuna	*T. alalunga*	*T. alalunga*
ALB-2	Albacore tuna	*T. alalunga*	*T. alalunga*
ALB-3	Albacore tuna	*T. alalunga*	*T. alalunga*
ALB-4	Albacore tuna	*T. alalunga*	*T. alalunga*
ALB-5	Albacore tuna	*T. alalunga*	*T. alalunga*
YFT-1	Yellowfin tuna	*T. albacares*	*T. albacares*
YFT-2	Yellowfin tuna	*T. albacares*	*T. albacares*
YFT-3	Yellowfin tuna	*T. albacares*	*T. albacares*
YFT-4	Yellowfin tuna	*T. albacares*	*T. albacares*
YFT-5	Yellowfin tuna	*T. albacares*	*T. albacares*
YFT-6	Yellowfin tuna	*T. albacares*	*T. albacares*
YFT-7	Yellowfin tuna	*T. albacares*	*T. albacares*

YFT-8	Yellowfin tuna	*T. albacares*	*T. albacares*
YFT-9	Yellowfin tuna	*T. albacares*	*T. albacares*
YFT-10	Yellowfin tuna	*T. albacares*	*T. albacares*
YFT-11*	Yellowfin tuna	*T.obesus*	*T. obesus*
YFT-12	Yellowfin tuna	*T. albacares*	*T. albacares*
YFT-13	Yellowfin tuna	*T. albacares*	*T. albacares*
YFT-14	Bluefin tuna	*T. albacares*	*T. albacares*
LQ-1	Bluefin tuna	*T. thynnus*	*T. thynnus*
LQ-2	Bluefin tuna	*T. thynnus*	*T. thynnus*
LQ-3	Bluefin tuna	*T. thynnus*	*T. thynnus*
LQ-4	Bluefin tuna	*T. thynnus*	*T. thynnus*
LQ-5	Bluefin tuna	*T. maccoyii*	*T. maccoyii*
LQ-6	Bluefin tuna	*T. maccoyii*	*T. maccoyii*
LQ-7	Bluefin tuna	*T. thynnus*	*T. thynnus*
LQ-8	Bluefin tuna	*T. maccoyii*	*T. maccoyii*
LQ-9	Bluefin tuna	*T. maccoyii*	*T. maccoyii*
Lq-10*	Bluefin tuna	*T. albacares*	*T. albacares*
LQ-11	Bluefin tuna	*T. thynnus*	*T. thynnus*
LQ-12	Bluefin tuna	*T. thynnus*	*T. thynnus*
LQ-13	Bluefin tuna	*T. thynnus*	*T. thynnus*
LQ-14	Bluefin tuna	*T. maccoyii*	*T. maccoyii*
LQ-15*	Bluefin tuna	*T. albacares*	*T. albacares*
LQ-16	Bluefin tuna	*T. maccoyii*	*T. maccoyii*

[1]Note: "*" sample nominal is inconsistent with the result of identification.
